# Genome sequence of a native-feather degrading extremely thermophilic *Eubacterium, Fervidobacterium islandicum* AW-1

**DOI:** 10.1186/s40793-015-0063-4

**Published:** 2015-09-29

**Authors:** Yong-Jik Lee, Haeyoung Jeong, Gun-Seok Park, Yunyoung Kwak, Sang-Jae Lee, Sang Jun Lee, Min-Kyu Park, Ji-Yeon Kim, Hwan Ku Kang, Jae-Ho Shin, Dong-Woo Lee

**Affiliations:** School of Applied Biosciences, Kyungpook National University, Daegu, Korea; Super-Bacteria Research Center, Korea Research Institute of Bioscience and Biotechnology (KRIBB), Daejeon, Korea; Major of Food Biotechnology, Silla University, Busan, Korea; Infection and Immunity Research Center, Korea Research Institute of Bioscience & Biotechnology (KRIBB), Daejeon, Korea; Department of Animal Resources Development, National Institute of Animal Science, Rural Development Administration (RDA), Cheonan, Korea

**Keywords:** Native feather, Keratin, Degradation, Extremophile, *Fervidobacterium islandicum* AW-1

## Abstract

*Fervidobacterium islandicum* AW-1 (KCTC 4680) is an extremely thermophilic anaerobe isolated from a hot spring in Indonesia. This bacterium could degrade native chicken feathers completely at 70 °C within 48 h, which is of potential importance on the basis of relevant environmental and agricultural issues in bioremediation and development of eco-friendly bioprocesses for the treatment of native feathers. However, its genomic and phylogenetic analysis remains unclear. Here, we report the high-quality draft genome sequence of an extremely thermophilic anaerobe, *F. islandicum* AW-1. The genome consists of 2,359,755 bp, which encodes 2,184 protein-coding genes and 64 RNA-encoding genes. This may reveal insights into anaerobic metabolism for keratin degradation and also provide a biological option for poultry waste treatments.

## Introduction

Keratin, a key structural material in feathers, skin, hair, nails, horns, and scales, is one of the most abundant proteins on earth, and it is a mechanically durable and chemically unreactive protein. Since feather keratin contains a high content of cysteine (~7 %) in its amino acid sequence, it has a strong and fibrous matrix through disulfide bonds. Such a highly rigid, strongly cross-linked, indigestible polypeptide has very limited industrial applications due to its rigidity and indigestibility, and is thus often considered a solid waste. In fact, more than 5 millions of tons of chicken feathers in poultry industry are generated globally every year, and such waste by-products can cause a serious solid waste problem [1, 2]. At present, most waste chicken feathers are disposed by burning, burying in landfills or recycling into low quality animal feed. However, these disposal methods are restricted due to increase in greenhouse gas emissions and environmental pollution. Many efforts aimed at meeting environmental performance criteria and renewable energy production are in progress to degrade poultry feathers to soluble peptides and amino acids for the use of fertilizers, animal feedstock, and soil conditioner [3]. Thus, development of a bioconversion process for degradation of feathers will provide considerable opportunities for industrial applications [4, 5]. In this regard, keratinolytic microorganisms have great importance in feather waste degradation and its use for improvement of livestock feed and production of hydrolysates. Hence, many microbial keratinases, differing from commonly known proteases (e.g., trypsin, pepsin and papain), have been sought to hydrolyze this recalcitrant polypeptide. Toward this aim, several keratin-degrading microorganisms, including *Bacillus licheniformis* PWD-1 [6], *Aspergillus fumigatus* [7], and *Streptomyces pactum*DSM 40530 [8] have been isolated and characterized. Nevertheless, the efficiency and feasibility of such bioprocesses is still limited in terms of practical applications, mainly due to the instability of enzyme activity, low yields of keratin degradation, and its long process time.

Previously, we isolated an extremely thermophilic bacterium from a geothermal hot spring in Indonesia [9]. When grown in TF medium supplemented with 0.8 % (w/v) of native chicken feathers, this bacterium could degrade native chicken feathers completely within 48 h at 70 °C under anaerobic conditions. Morphological, physiological and 16S rRNA gene sequencing analyses demonstrated that this native chicken feather degrading bacterium belonging to the genus *Fervidobacterium* was identified as *Fervidobacterium islandicum* AW-1 [9]. Moreover, it was found that adding the reducing reagent greatly hastened the degradation of native chicken feathers, indicating that breakage of disulfide bonds are also responsible for the complete degradation of feather keratin. Therefore, we hypothesized that not only keratinolytic proteases but also other enzymes specific to disulfide bonds might be mainly involved in degradation of keratin. Accordingly, these and related reasons led us to sequence the whole genome of *F. islandicum* AW-1, providing an insight into the degradation of non-digestible keratin biomass. Moreover, comparative genomics for feather-degrading *F. islandicum* AW-1 and its closely related non-degrading bacteria will shed light on the evolutionary relationship between them. Here, we present a summary of classification and a set of general features for *F. islandicum* AW-1 together with the description of genome properties and annotation.

## Organism information

### Classification and features

**Fig. 1:**
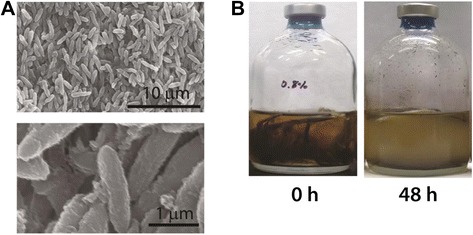
**a** The scanning electron micrographs (SEM) of *F. islandicum* AW-1 grown on the TF medium supplemented with glucose (0.5 %, w/v) during anaerobic fermentation at 70 °C. **b** Complete degradation of native feathers by *F. islandicum* AW-1. The cells were grown on the TF medium supplemented with native feathers (0.8 %. w/v) during anaerobic fermentation at 70 °C for 48 h. For the preparation of specimens for *F. islandicum* AW-1, we followed the protocol as described previously

**Fig. 2:**
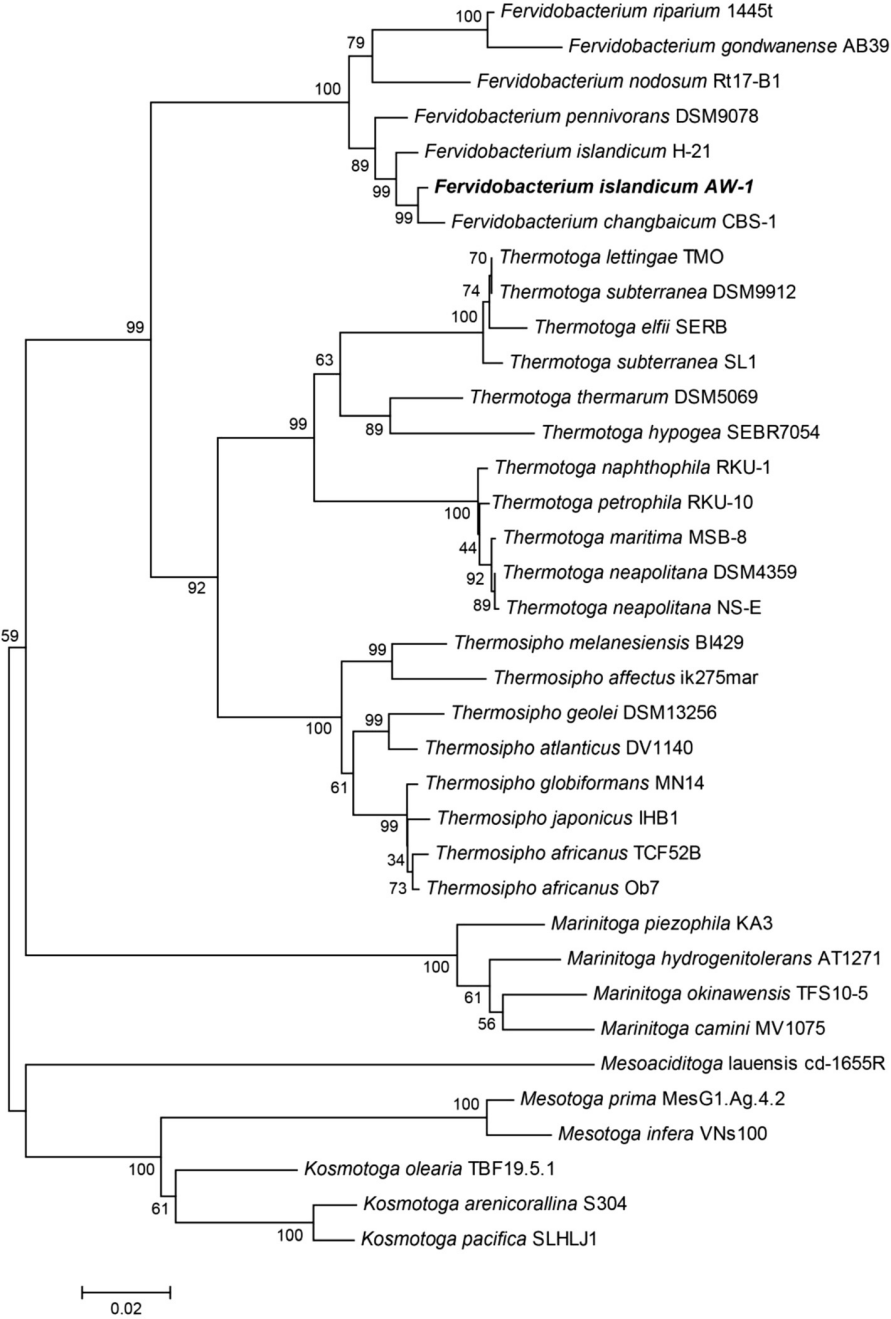
Phylogenetic tree based on 16S rRNA gene sequences showing the relationship of *F. islandicum* AW-1 (in bold) to members of the family *Thermotogaceae.* The evolutionary history was inferred using the Neighbor-Joining method. The analysis involved 36 nucleotide sequences. All positions containing gaps and missing data were eliminated. There were a total of 1,235 positions in the final dataset. Bootstrap values (percentages of 1,000 replications) are shown next to the branches. The sequences used in the analysis were obtained from the GenBank database. Bar, 2 nt substitution per 100 nt. Evolutionary analyses were conducted in MEGA6

Out of 37 native chicken feather-degrading anaerobic strains grown at 70 °C enriched in EM-1 medium supplemented with native chicken feathers as a carbon source, we chose the strain AW-1 showing the highest keratinolytic activity [9]. Subsequently, we identified the strictly anaerobic, rod shaped (0.6 × 1 ~ 3.5 μm), motile, non-sporulating, Gram-negative extremophilic bacterium as *Fervidobacterium islandicum* AW-1 based on cell morphology, physiological characteristics, common DNA characteristics, 16S rRNA gene sequence, and cellular fatty acid profile as described previously (Fig. 1a, b) [9]. This bacterium belongs to the order of *Thermotogales*, of which all members are Gram-negative rod-shaped anaerobic extremophiles containing unique lipids [10]. After the first isolate *F. nodosum* had been reported, several *Fervidobacterium* strains including *F. islandicum* [11], *F. gondwanense* [12], *F. pennivorans* [13], *F. changbaicum* [14], and *F. riparium* [15] were isolated and characterized. All of them grew on glucose, mainly producing H_2_, CO_2_, and acetate, and also fermented a wide range of nutrients such as peptone, yeast extract, pyruvate, glucose, maltose, raffinose, and starch. Such organotrophs can also reduce S^0^ to H_2_S during the course of fermentation. In particular, *F. islandicum* AW-1 showed the highest keratinolytic activity, resulting in the complete degradation of native chicken feathers (8 g/L) within 48 h (Fig. 1b), and its optimal growth temperature and pH on the native feathers were 70 °C and pH 7.0, respectively [9]. Among the genus *Fervidobacterium*, *F. islandicum* AW-1 together with *F. pennivorans* have been found as native-feather degrading bacteria [9, 13]. Fig. 2 shows the phylogenetic neighborhood of *F. islandicum* AW-1 in a 16S rRNA gene sequence-based tree. This strain clusters closest to the genus of *Fervidobacterium*, the *Thermotogales* order. The 16S rRNA gene sequence (1456 bp) of *F. islandicum* AW-1 obtained from its genome sequence showed high levels of sequence similarity with members of the genus *Fervidobacterium*, such as *F. changbaicum* (99.3 %) [14], *F. pennivorans* (98.1 %) [13], *F. islandicum* (97.3 %) [11], *F. riparium* (96.1 %) [15], *F. gondwanense* (94.7 %) [12] and *F. nodosum* (95.4 %) [16] (Fig. 2). RAST analysis to rapidly call and annotate the genes of a complete or essentially complete prokaryotic genome [17] also suggested that *F. nodosum* Rt17-B1 was actually *F. islandicum* AW-1's closest neighbor. ANI analysis using BLAST [18] showed that, among the completely sequenced *Fervidobacterium* and *Thermotoga* species, *F. pennivorans* was closest to *F. islandicum* AW-1 (77.4 % sequence identity and 78.9 % alignment). As shown in Fig. 1, this strain was rod-shaped, occurring singly, in pairs or short chains with a single polar spheroid, a sheath-like outer membrane structure, a so called “toga”, which is a typical morphological feature belonging to the order of *Thermotogales*. Together with the previous phenotypic and biochemical characterization [9], our sequence analysis suggested that this AW-1 strain could be assigned as a native feathers degradable strain of *F. islandicum*. This was also supported by the previous DNA-DNA hybridization analysis with *F. islandicum* (92.4 %) [11] and *F. pennivorans* (42 %) [13].

## Genome sequencing information

### Genome project history

This bacterium was selected for sequencing to unveil the degradation mechanism of keratin through transcriptomic analysis and comparative genomics based on its ability to completely decompose native feathers under anaerobic conditions at elevated temperatures (Table 1, Fig. 1b). The next-generation sequencing was performed at Pacific Biosciences (Menlo Park, CA). The assembly and annotation were performed by using the hierarchical genome-assembly process [19] protocol RS HGAP Assembly 2 in SMRT analysis version 2.2.0 (Pacific Biosciences), NCBI COG [20] and RAST server database [17]. The whole complete genome sequence of *F. islandicum* AW-1 has been deposited at DDBJ/EMBL/GenBank under the accession number. The AW-1 strain is also available from the Korean Collection for Type Cultures (KCTC, Daejeon, Korea). A summary of the project information is shown in Table 2.

**Table 1:** Classification and general features of *Fervidobacterium islandicum* AW-1 [29]

MIGS ID	Property	Term	Evidence code^a^
	Classification	Domain *Bacteria*	TAS [30]
		Phylum *Thermotogae*	TAS [31, 32]
		Class *Thermotogae*	TAS [31, 33]
		Order *Thermotogales*	TAS [31, 34]
		Family *Fervidobacteriaceae*	TAS [31]
		Genus *Fervidobacterium*	TAS [31, 16]
		Species *Fervidobacterium islandicum*	TAS [11]
		(Type) strain: AW-1	TAS [9]
	Gram stain	Negative	TAS [9]
	Cell shape	Rod	TAS [9]
	Motility	Motile	TAS [9]
	Sporulation	Non-sporulatings	TAS [9]
	Temperature range	40-80 °C	TAS [9]
	Optimum temperature	70 °C	TAS [9]
	pH range; Optimum	5.0 ~ 9.0; 7	TAS [9]
	Carbon source	Varied	TAS [9]
MIGS-6	Habitat	Geothermal hot stream	TAS [9]
MIGS-6.3	Salinity	Not reported	
MIGS-22	Oxygen requirement	Anaerobic	TAS [9]
MIGS-15	Biotic relationship	Free-living	TAS [9]
MIGS-14	Pathogenicity	Not reported	
MIGS-4	Geographic location	Indonesia/Sileri	TAS [9]
MIGS-5	Sample collection	August, 1999	NAS
MIGS-4.1	Latitude	Not recorded	
MIGS-4.2	Longitude	Not recorded	
MIGS-4.4	Altitude	Not recorded	

^a^Evidence codes - *IDA* Inferred from Direct Assay, *TAS* Traceable Author Statement (i.e., a direct report exists in the literature), *NAS* Non-traceable Author Statement (i.e., not directly observed for the living, isolated sample, but based on a generally accepted property for the species, or anecdotal evidence). These evidence codes are from the Gene Ontology project [35]

**Table 2:** Project information

MIGS ID	Property	Term
MIGS 31	Finishing quality	Improved-high-quality draft
MIGS-28	Libraries used	10 kb SMRT library
MIGS 29	Sequencing platforms	PacBio RS II
MIGS 31.2	Fold coverage	351.41 ×
MIGS 30	Assemblers	RS HGAP assembly protocol in SMRT analysis pipeline v.2.2.0
MIGS 32	Gene calling method	NCBI prokaryotic genome annotation pipeline, genemarkS
	Locus Tag	NA23
	Genbank ID	JRRD00000000.2
	Genbank date of release	December 04, 2014
	GOLD ID	Gp0109425
	BIOPROJECT	PRJNA263006
MIGS 13	Source material identifier	KCTC 4680
	Project relevance	Environmental, bioremediation, biodegradation, biotechnological

### Growth conditions and genomic DNA preparation

*F. islandicum* AW-1 was grown in TF medium which contained the following: 0.5 % glucose (instead of 0.8 % native chicken feather), 1 g of yeast extract, 1.6 g of K_2_HPO_4_, 0.8 g of NaH_2_PO_4_ · H_2_O, 0.16 g of MgSO_4_ · 7H_2_O, 0.1 g of NH_4_Cl, 1 % (v/v) vitamin solution (2 g of biotin, 2 g of folic acid, 10 g of pyridoxine-HCl, 5 g of thiamine-HCl, 5 g of riboflavin, 5 g of nicotinic acid, 5 g of calcium pantothenate, 0.1 g of vitamin B_12_, 5 g of p-aminobenzoic acid, 5 g of lipoic acid per liter), 1 % (v/v) trace element solution (2 g of nitrilotriacetic acid, 0.18 g of ZnSO_4_ · 7H_2_O, 3 g of MgSO_4_ · 7H_2_O, 0.5 g of MnSO_4_ · 2H_2_O, 1 g of NaCl, 0.1 g of FeSO_4_ · 7H_2_O, 0.01 g of H_3_BO_3_, 0.18 g of CoSO_4_ · 7H_2_O, 0.01 g of CuSO_4_ · 5H_2_O, 0.1 g of CaCl_2_ · 2H_2_O, 0.1 g of AlK(SO_4_)_2_ · 12H_2_O, 0.001 g of Na_2_SeO_3_ · 5H_2_O, 0.025 g of NiCl_2_ · 6H_2_O, 0.01 g of Na_2_MoO_4_ · 2H_2_O per liter), 1 mg of resazurin and 0.75 g of Na_2_S · 9H_2_O per liter at pH 7 and 70 °C. The media were prepared as follows; under the N_2_ gas flushing, adjusted to 7 with 2 N HCl (NaOH), and sterilized by autoclaving at 121 °C for 20 min prior to use [9]. The genomic DNA was isolated from a 12 h-grown cells (5 ~ 7 × 10^8^ cells/ml) in TF medium (0.5 L) using a QIAmp DNA mini kit (QIAGEN).

### Genome sequencing and assembly

**Fig. 3:**
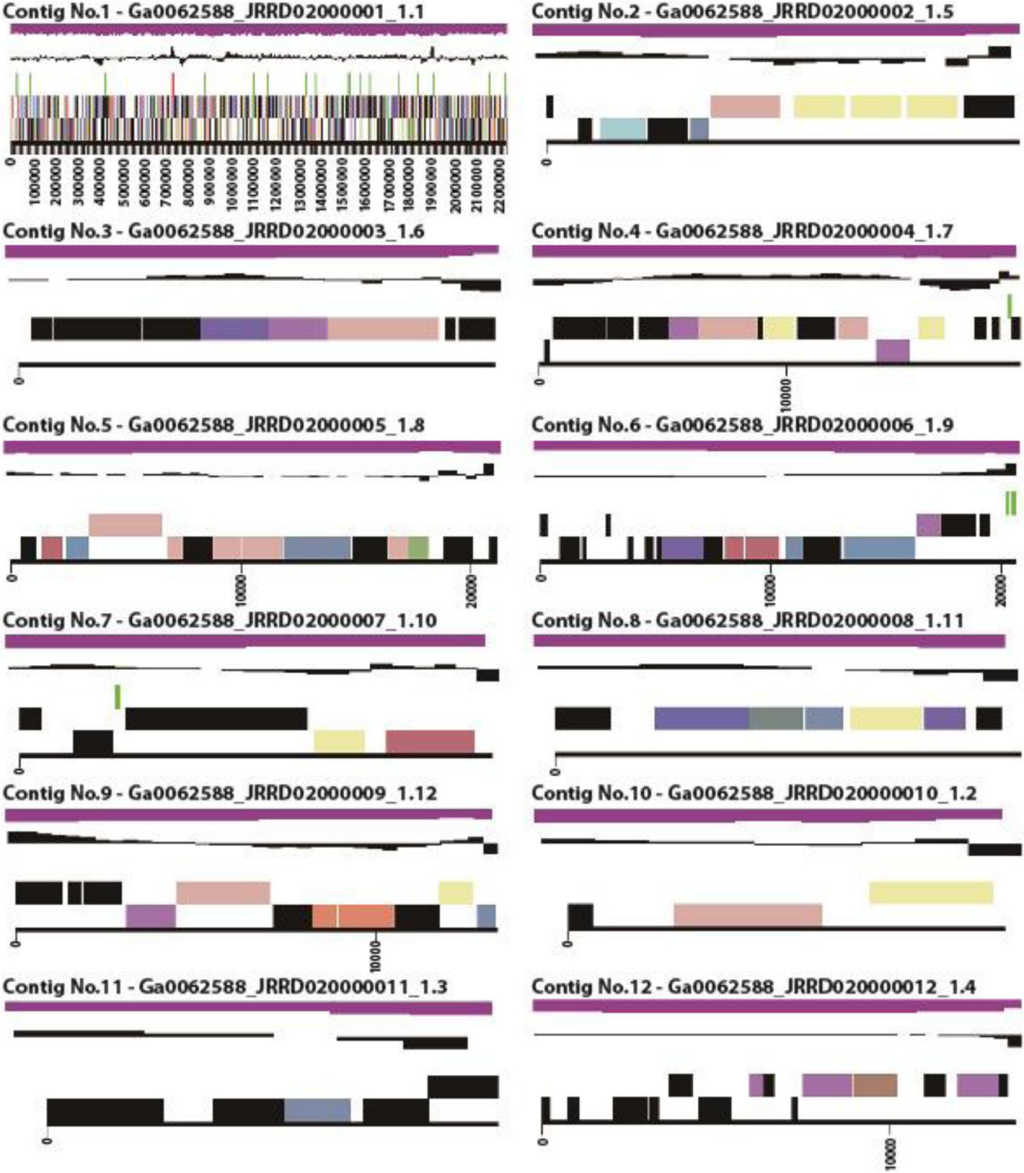
Graphical linear map of the genome of *F. islandicum* AW-1 strain. From the bottom to the top of each scaffold: Genes on the forward strand (color by COG categories as denoted by the IMG platform), Genes on the reverse strand (color by COG categories), RNA genes (tRNAs green, sRNAs red, other RNAs black), GC content, GC skew

Genome sequencing was performed using a single molecule real-time sequencing platform on PacBio RS II instrument with P4-C2 chemistry (Pacific Biosciences, Menlo Park, CA) [21]. Preprocessing of reads and *de novo* assembly were performed using the hierarchical genome-assembly process [19] protocol RS HGAP Assembly 2 in SMRT analysis version 2.2.0 (Pacific Biosciences). Standard parameters were applied as follows: PreAssembler v2 (Minimum Seed Read Length : 6,000 bp) was conducted then Celera Assembler v1 (Genome Size : 2,500,000 bp, Target Coverage : 30, Overlapper Error Rate : 0.06, Overlapper Min Length : 40, Overlapper K-mer : 14) was performed [19]. We assembled 169,795 reads (achieving ~351.41 fold coverage) into 12 contigs over 2,000 bp. The total contig length, maximum contig size, average contig length, and *N*_50_ were 2,359,755 bp, 2,232,638 bp, 196,624 bp, and 2,232,638 bp, respectively (40.74 % G + C) (Fig. 3 and Table 3).

**Table 3:** Genome statistics

Attribute	Value	% of Total
Genome size (bp)	2,359,755	100.00
DNA coding (bp)	2,156,275	91.38
DNA G + C (bp)	961,311	40.74
DNA scaffolds	12	100.00
Total genes	2,248	100.00
Protein coding genes	2,184	97.15
RNA genes	64	2.85
Pseudo genes	75	3.34
Genes in internal clusters	228	10.14
Genes with function prediction	1,823	81.09
Genes assigned to COGs	1,512	67.26
Genes with Pfam domains	1,842	81.94
Genes with signal peptides	44	1.96
Genes with transmembrane helices	658	29.27
CRISPR repeats	2	0.09

### Genome annotation

The genes in the assembled genome were annotated using NCBI COG [20]. Additionally, automatic functional annotation of genes was conducted using the RAST server database [17]. Genes were predicted using GeneMarkS [22] as a part of the NCBI prokaryotic genome automatic annotation pipeline (PGAAP) [23]. Besides functional annotation for protein coding genes, PGAAP also provided information for RNA genes and pseudo genes. BLASTCLUST parameters for identifying internal clusters were ‘-L .8 –b T –S 50’. Proteins with Pfam domains, signal peptides, and transmembrane helices were identified using InProScan search against HMMPfam [24], SignalPHMM [25], TMHMM [26] via Blast2Go service [27]. Additional gene prediction and functional annotation were carried out using Integrated Microbial Genomes (IMG-ER) platform [28].

## Genome properties

The total size of the genome is 2,359,755 bp, slightly larger than those of other sequenced *Fervidobacterium* strains and G + C content is 40.7 % (Table 3). A total of 2,184 protein coding genes were predicted in 2,248 total numbers of genes, indicating that 64 RNAs sequences were identified and 361 of protein coding genes were assigned to a putative function with the remaining annotated as hypothetical proteins. The detailed properties and the statistics of the genome as well as the distribution of genes into COG functional categories are summarized in Tables 3 and 4.

**Table 4:** Number of genes associated with general COG functional categories

Code	Value	% age	Description
J	138	6.32	Translation, ribosomal structure and biogenesis
A	0	0.00	RNA processing and modification
K	73	3.34	Transcription
L	140	6.41	Replication, recombination and repair
B	1	0.05	Chromatin structure and dynamics
D	18	0.82	Cell cycle control, Cell division, chromosome partitioning
V	23	1.05	Defense mechanisms
T	64	2.93	Signal transduction mechanisms
M	74	3.39	Cell wall/membrane biogenesis
N	59	2.70	Cell motility
U	35	1.60	Intracellular trafficking and secretion
O	59	2.70	Posttranslational modification, protein turnover, chaperones
C	105	4.81	Energy production and conversion
G	168	7.69	Carbohydrate transport and metabolism
E	142	6.50	Amino acid transport and metabolism
F	54	2.47	Nucleotide transport and metabolism
H	60	2.75	Coenzyme transport and metabolism
I	37	1.69	Lipid transport and metabolism
P	92	4.21	Inorganic ion transport and metabolism
Q	16	0.73	Secondary metabolites biosynthesis, transport and catabolism
R	185	8.47	General function prediction only
S	119	5.45	Function unknown
-	736	33.70	Not in COGs

The total is based on the total number of protein coding genes in the genome

## Insights from the genome sequence

**Fig. 4:**
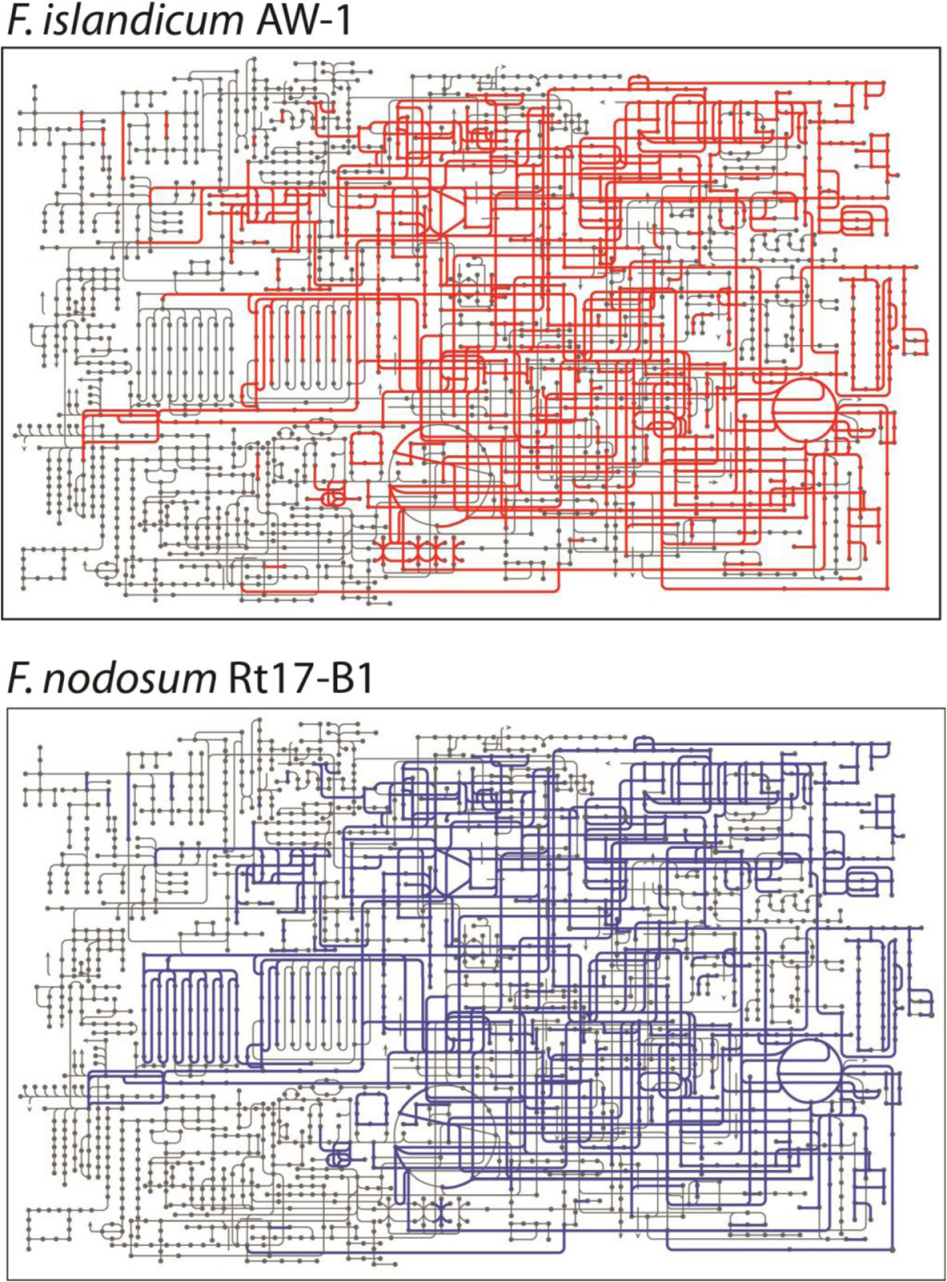
Overview of the microbial pathways on the KEGG pathways using the iPath. Metabolic pathways found in the context of *F. islandicum* AW-1 (top panel) and *F. nodosum* Rt17-B1 (bottom panel) genomes are shown in red and blue, respectively. Hypothetical proteins found are excluded

As described above, the 16S rRNA gene sequence of *F. islandicum* AW-1 showed the high similarity to those of *F. changbaicum*CBS-1, and *F. islandicum* H-21. On the other hand, RAST analysis demonstrated that *F. nodosum* Rt17-B1 was actually *F. islandicum* AW-1's closest neighbor. Consequently, genome analysis found genes involved in protein metabolism including protein degradation systems with 25 different types of proteases. For example, protein-coding genes annotated as carboxyl-terminal protease (EC 3.4.21.102) and lipoprotein signal peptidase (EC 3.4.23.36) were found in *F. islandicum* AW-1, but not in *F. nodosum* Rt17-B1. We also found several genes encoding cysteine desulfurase and thioredoxin-disulfide reductase as potential candidates for feather degradation. In addition, several reductases and peptidases (e.g., disulfide reductase, thioredoxin, and carboxy-peptidases) of *F. islandicum* AW-1 showed relatively low levels of sequence identity (less than 50 %) to those of *F. nodosum* Rt17-B1. In addition, *F. islandicum* AW-1 seems to have several distinct enzymes involved in amino-sugars (chitin and *N*-acetylglucosamine) utilization and sugar alcohols (glycerol and glycerol-3-phosphate) metabolism, which are not found in *F. nodosum* Rt17-B1 (Fig. 4). Notably, comparative analysis of the *F. islandicum* AW-1 and *F. nodosum* RT17-B1 genomes revealed that the former seems to have several distinct enzymes involved in fatty acid degradation, aromatic compound degradation, and alpha-linolenic acid metabolism not found in the latter.

Previously, it was found that addition of the reducing reagent greatly hastened the degradation of native feathers, indicating that breakage of disulfide bonds are also responsible for the complete degradation of feather keratin, implying that not only keratinolytic proteases but also other enzymes specific to disulfide bonds might be mainly involved in degradation of keratin [9]. Indeed, comparison of the genome sequence of *F. islandicum* AW-1 with that of *F. nodosum* Rt17-B1 suggests that several candidate enzymes including cysteine desulfurase and thioredoxin-disulfide reductase may be involved in native feather degradation. In addition, the genome of *F. islandicum* AW-1 reveals that this strain also possesses some hydrogenases. Therefore, *F. islandicum* AW-1 may provide a biological option for biohydrogen production as well as poultry waste treatments.

## Conclusions

Among the genus of *Fervidobacterium*, *F. islandicum* AW-1 and *F. pennivorans* have been found as native-feather degrading bacteria [13, 9]. Compared to other *Fervidobacterium* strains, the genome-based approach for this extremely thermophilic bacterium is of great importance and interest not only for keratin degradation, but also for elucidation of distinct amino acid and carbohydrate metabolic pathways. Accordingly, these and related reasons led us to sequence the whole genome of *F. islandicum* AW-1, providing an insight into the degradation of non-digestible keratin biomass. Moreover, comparative genomics for feather-degrading *F. islandicum* AW-1 and its closely related non-degrading bacteria will shed light on the evolutionary relationships among them. Overall, this genomic analysis may provide not only an insight into the mechanism of keratin degradation, but also an industrial option applicable for the treatment of non-digestible biomass.

## Abbreviations

RAST: Rapid Annotation using Subsystem Technology
iPath: Interactive Pathway Explorer
KEGG: Kyoto Encyclopedia of Genes and Genomes
